# Apophysitis of the Tibial Tuberosity (Osgood-Schlatter Disease): A Review

**DOI:** 10.7759/cureus.780

**Published:** 2016-09-13

**Authors:** Raju Vaishya, Ahmad Tariq Azizi, Amit Kumar Agarwal, Vipul Vijay

**Affiliations:** 1 Orthopaedics, Indraprastha Apollo Hospitals; 2 Orthoapedics, Herat Regional Hospital, Herat, Afghanistan

**Keywords:** tibial tuberosity, osgood-schlatter disease, apophysitis

## Abstract

Osgood-Schlatter disease (OSD) is a condition in which the patellar tendon insertion on the tibial tuberosity becomes inflamed. It is a well-known condition in late childhood characterized by pain and a bony prominence over the tibial tuberosity. The pain is usually exacerbated by physical activities like running, jumping, and climbing stairs. In the acute stage, the margins of the patellar tendon become blurred in radiographs due to the soft tissue swelling. After three to four months, bone fragmentation at the tibial tuberosity is viewed. In the sub-acute stage, soft tissue swelling resolves, but the bony ossicle remains. In the chronic stage, the bone fragment may fuse with the tibial tuberosity which can appear normal.

The primary goal in the treatment of OSD is the reduction of pain and swelling over the tibial tuberosity. The patient should limit physical activities until the symptoms are resolved. In some cases, the patient should restrict physical activities for several months. The presence of pain with kneeling because of an ossicle that does not respond to conservative measures is the indication for surgery. In these cases, the removal of the ossicle, surrounding bursa, and the bony prominence is the treatment of choice.

## Introduction and background

Osgood-Schlatter disease (OSD, also called Lannelongue’s disease) is a condition in which the patellar tendon insertion on the tibial tuberosity becomes inflamed [1-2]. It is a well-known condition in late childhood characterized by pain over the tibial tuberosity along with a bony prominence. Usually, it resolves at the end stages of skeletal growth. Boys are affected more often than girls, and the age of affection is typically in the second decade of life (10 to 15 years in boys and eight to twelve years in girls) [3]. Usually, less than 25% of patients complain of pain over the tibial tuberosity. In the early stages of OSD, the patients have pain on the tibial tuberosity after physical activities, but over time, the pain becomes permanent and steady regardless of activity. In x-rays, a regular ossification (ossicle) is demonstrated over the tibial tuberosity. Treatment includes conservative and surgical options. Conservative treatment includes modifying physical activities, using ice packs, nonsteroidal anti-inflammatory drugs (NSAIDs), braces, and pads. Symptoms usually resolve after the closure of the physis without any treatment, but symptoms may remain in some cases. In almost ten percent of patients, the bone fragments do not fuse, and these patients complain of pain in front of the knee, even after slight physical activity but especially after kneeling [4-6]. This pain typically relates to the mobile and unfused bone fragments, which may require surgical excision.

## Review

### Clinical Presentation

**Figure 1:**
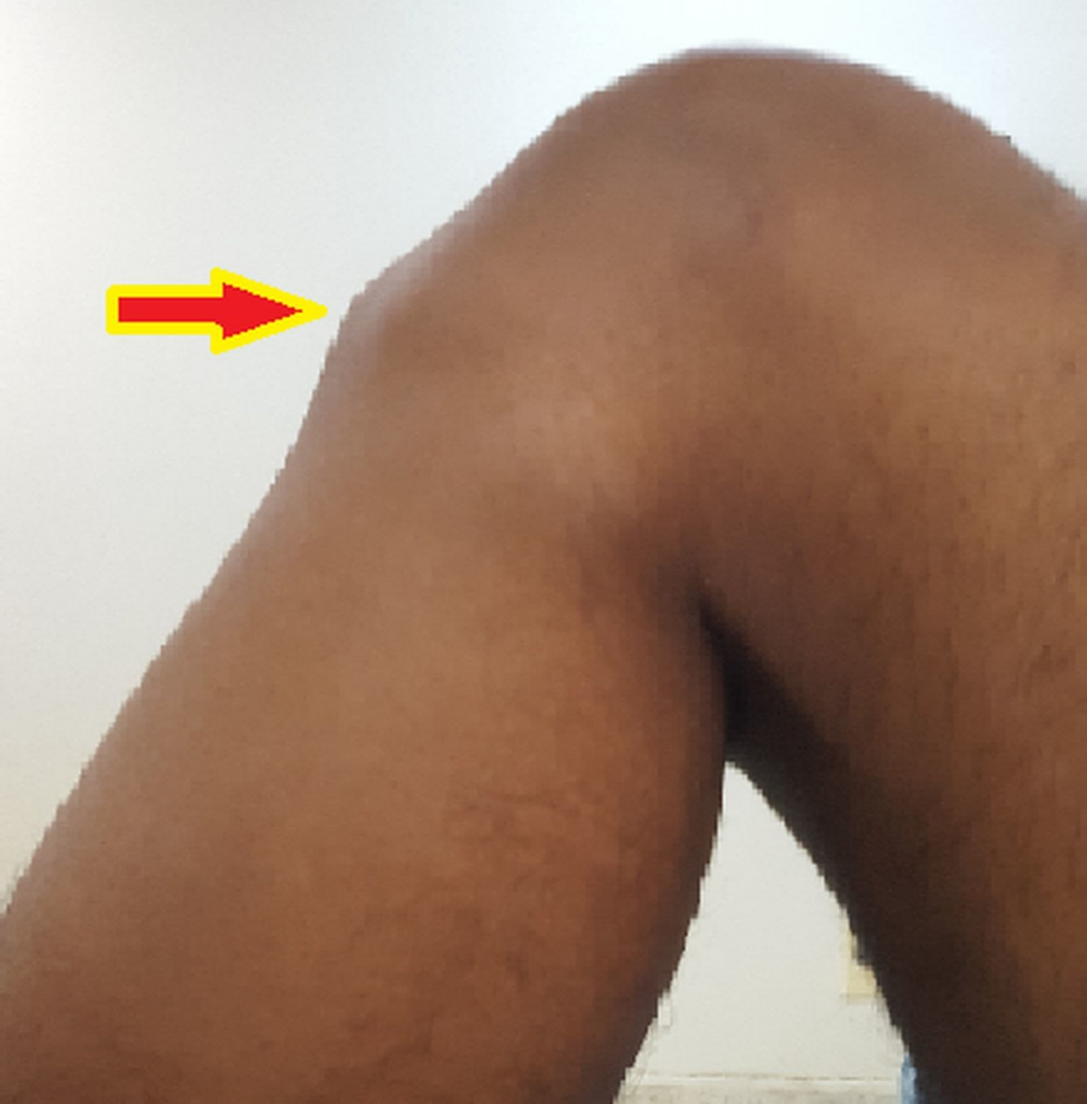
Bony prominence over the tibial tuberosity in OSD

Pain and swelling are the primary symptoms felt in the lower aspect of the knee, around the patellar attachment to the tibial tuberosity (Figure 1) [7-8].

The pain is usually exacerbated by physical activities like running, jumping, and climbing stairs. Knee extension against resistant and other sports-related activities may worsen the symptoms. In 20% to 30% of cases, both knees are affected, but symptom intensity can vary in each knee.

### Investigations

**Figure 2:**
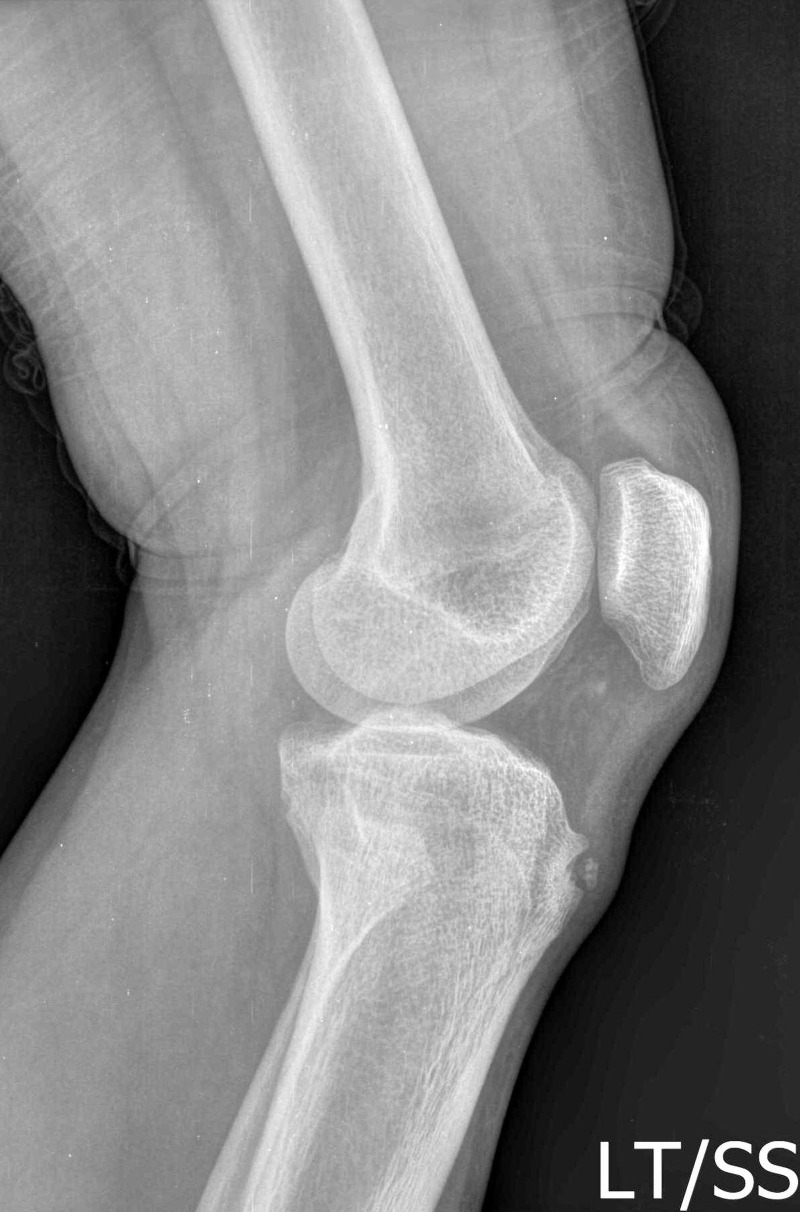
Separated ossicle and bony prominence seen in lateral radiography of knee in OSD.

In the acute stage, the margins of the patellar tendon become blurred in radiographs due to the soft tissue swelling. After three to four months, bone fragmentation at the tibial tuberosity is viewed. In the sub-acute stage, soft tissue swelling resolves, but the bone ossicle remains. In the chronic stage, the bone fragment may fuse with the tibial tuberosity which can appear normal. However, sometimes the bone fragment is displaced (Figure 2).

Ultrasound may be useful as it determines any swelling in the soft tissue, cartilage, bursa, and tendon [9-10]. It also detects any new bone formation if present in the area. Magnetic resonance imaging is more sensitive than ultrasound and determines soft tissue swelling in front of the tibial tuberosity, edema of the patellar tendon, infrapatellar bursitis (a deep infrapatellar bursitis is a frequent finding) [11], and bone marrow edema.

### Differential diagnosis

**Figure 3:**
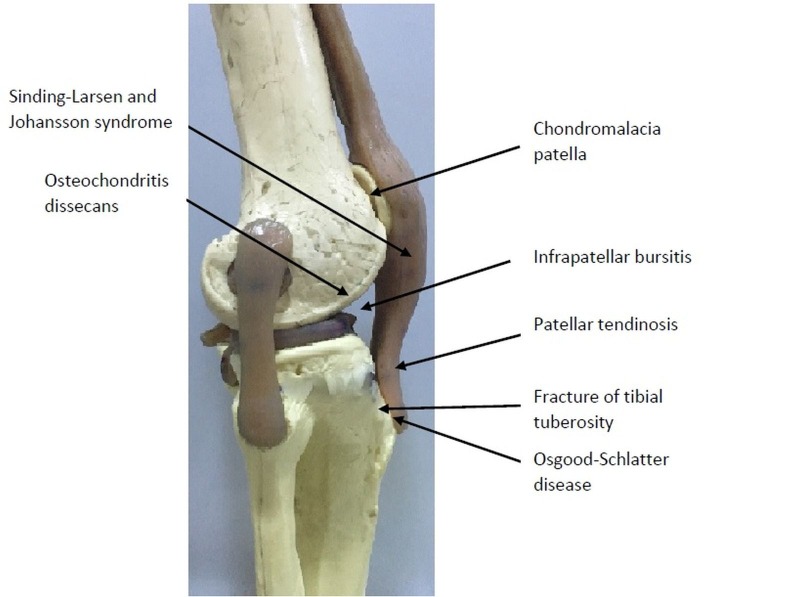
Other conditions that should be considered in differential diagnosis of OSD have been marked over the bone model

There are some other diseases that should be considered in the differential diagnosis of OSD, such as Sinding-Larsen-Johansson syndrome, Hoffa’s syndrome, soft tissue or bone tumors, patellar tendon avulsion or rupture, chondromalacia patella, patellar tendinitis, infectious apophysitis, accessory ossification centers, osteomyelitis of the proximal tibia, and tibial tubercle fracture (Table 1, Figure 3) [12].

**Table 1:** Table showing the differential diagnosis of OSD

Condition	Signs/symptoms	Investigations
Fracture of tibial tuberosity	A history of trauma is present, the onset of symptoms is sudden, and the patient is not able to extend the knee or bear weight on the knee.	An irregular line is present on x-ray without fragmentation of tibial tuberosity.
Hoffa’s disease (Fat pad hypertrophy/impingement)	Tenderness in the anterior joint line lateral to the patellar tendon.	X-ray is normal in Hoffa’s disease.
Sinding-Larsen and Johansson syndrome (Inferior patellar pole traction apophysitis)	Maximal tenderness is at the inferior pole of the patella, not at the tibial tubercle.	On x-ray, the tibial tuberosity is normal, and an ossicle or osteophyte in the lower pole of the patella is present.
Infrapatellar bursitis	It is difficult to differentiate infrapatellar bursitis from OSD clinically; the location of pain is at or near the attachment of the patellar tendon to the tibial tuberosity, but there may be no tenderness when palpating the tibial tuberosity.	X-ray is normal or may show a soft tissue swelling. In MRI, tibial tuberosity is normal, but it shows the fluid collection in the infrapatellar region.
Osteomyelitis	Pain may be present with activity or rest, and systemic symptoms and signs of infection are present.	In blood exam, there are increased levels of ESR, CRP, and WBC. Blood culture is positive, and soft tissue swelling periosteal reaction are seen in x-ray.
Osteochondritis dissecans of the knee	Pain is located in the anterior or anteromedial aspect of the knee. Tenderness is localized to the joint line (usually medial), with no tenderness on tibial tuberosity.	The lesion is apparent via x-ray in the lateral aspect of the medial femoral condyle. Otherwise, an MRI is needed for diagnosis.
Patellar tendonitis	It is difficult to differentiate from OSD, and may occur as a complication of OSD.	Radiographic studies are normal or may show a soft tissue swelling. Tibial tuberosity appears normal in MRI or may show increased signal in the patellar tendon.
Chondromalacia patella (runner’s knee)	Pain is present in the knee region (patellofemoral pain). On examination, pain becomes apparent with pressure on patella or manipulating patella above femoral condyle [14]. A grinding or cracking feel is present during extension and flexion of the knee joint.	In the radiographic study, there may be bone damage, or signs of arthritis seen. MRI will reveal any cartilage damage.

### Management

The primary goal in the treatment of OSD is the reduction of pain and swelling over the tibial tuberosity. For this reason, the patient should limit physical activities until the symptoms are resolved, which can sometimes take several months. Other conservative treatments include the use of ice packs, NSAIDs like ibuprofen and naproxen (to reduce pain and swelling), protective padding, and physiotherapy.

Physiotherapy

Exercises for the improvement of the quadriceps, hamstrings, and gastrocnemius muscles are recommended. Immobilizing the knee (using a cast or brace) can reduce strain on the tibial tuberosity [13,14].

Surgery

Surgery is indicated when ossicle pain on kneeling persists despite conservative treatment measures. In these cases, removal of the ossicle, surrounding bursa, and the bony prominence is the treatment of choice [15]. For a growing child, surgery does not seem to offer any benefit. A five-year follow-up trial reviewed two groups of patients with these symptoms. One group was treated surgically by tibial sequestrectomy, and the other group was treated conservatively. No benefit was found in the first method versus the second [16]. Binazzi, et al. found that the excision of the ossicle is the standard surgical method and removal of the prominent tibial tuberosity is optional [17].

Pihlajamäki, et al. studied the outcomes of the patients who did not respond to conservative measures and were surgically treated [18]. In a ten-year study period, they found that 93 patients (87%) participated in daily activities normally, 80 (75%) patients reached their pre-surgery level of sports activity. Forty-one patients (38%) could kneel without restriction or pain. Mild postsurgical complications were seen in six patients.

**Figure 4:**
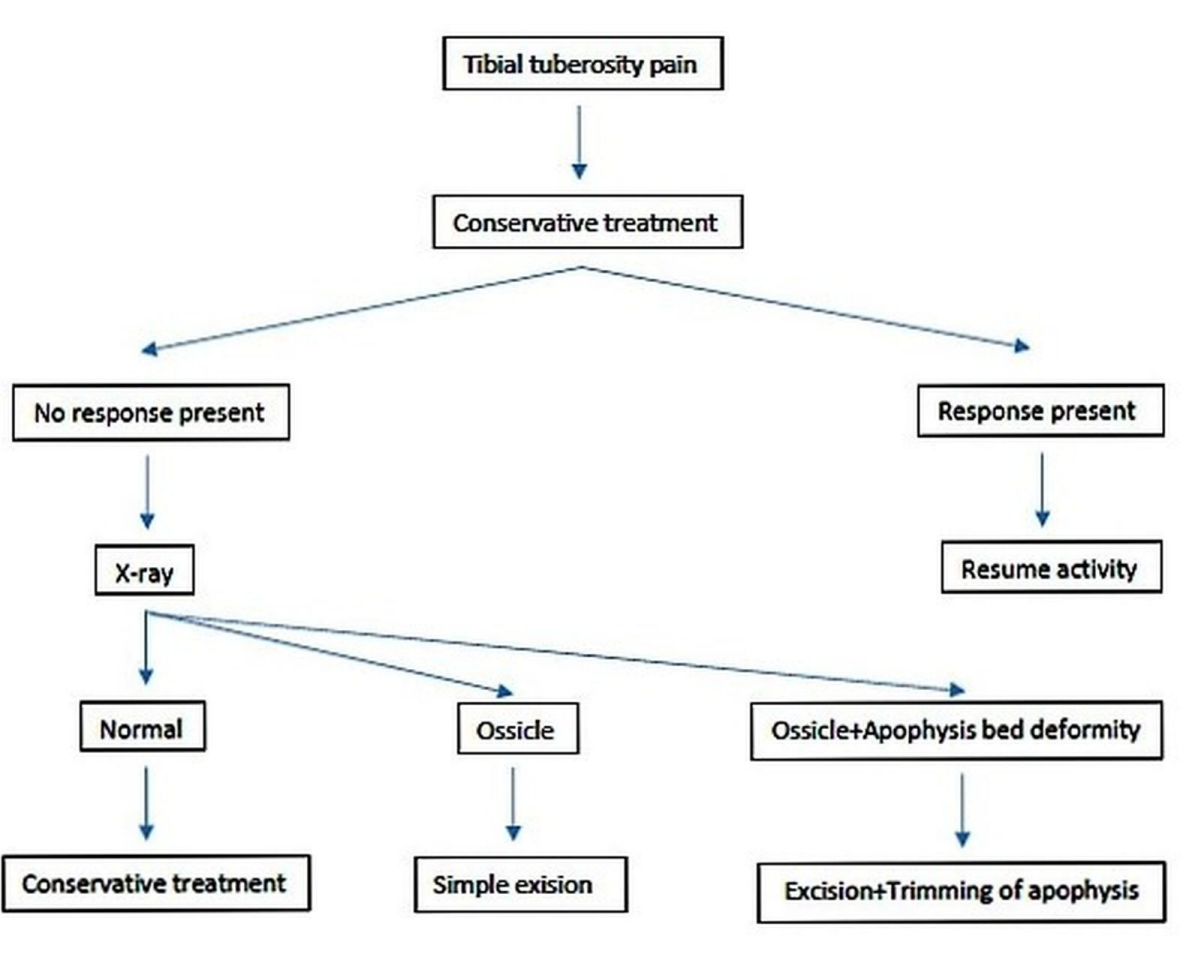
The algorithm for treatment of OSD

The detailed algorithm for treatment of OSD is presented in Figure 4.

Rehabilitation

In this period, the focus is on muscle strengthening, gait training, and pain control to restore a functional knee.

### Discussion

OSD is usually a self-limiting disease, and symptoms may resolve without any particular treatment or with simple conservative treatment, like activity restriction or immobilization in a cast for three to six weeks. Surgery is rarely indicated for OSD. Krause, et al. studied the natural history of untreated OSD. In 69 affected knees in 50 patients, they found that 76% of patients had no activity restriction, but only 60% of patients [PME1] could kneel without pain [19]. They suggested that persistent symptoms of OSD for more than two years warranted exploration. Krause, et al. noted that symptoms of OSD are resolvable in most of the cases, and, in those cases that persist, x-rays may show a fragmentation of the tibial tuberosity. Lynch and Walsh described that among the patients they followed, just two patients had premature fusion of the tibial apophysis with conservative treatment [6].

If the symptoms persist, surgery may be indicated. One trial noted, however, that after tibial sequestrectomy (removal of the fragments), clinical results were no better than those seen with conservative treatments [20]. Bosworth recommended inserting bone pegs into the tibial tubercle; this is a simple procedure and usually, resolves the symptoms [20]. Thomson and Ferciot, et al. pointed out, however, that an indecent prominence remains after this surgery [21-22]. They recommended a longitudinal incision in the patellar tendon for the excision of the bony prominence. Longitudinal growth of the tibia was not disturbed in a series of 41 operations by Thomson, et al., 11 in the Ferciot series, and 42 in the Flowers and Bhadreshwar series [21-22,5]. The reported complications of OSD in the patients treated conservatively or surgically, include patellar subluxation, patella alta, non-fusing fragments or a premature fusion that could result in genu recurvatum. Høgh and Lund recommended delaying surgery until the apophysis has fused [23].

## Conclusions

Osgood-Schlatter disease (OSD) is usually a self-limiting disease mainly seen in late childhood. The symptoms usually resolve without any specific treatment or with simple conservative treatment such as the restriction of activity or immobilization in a cast for three to six weeks. Surgery is rarely indicated for OSD. If the symptoms persist and become disabling to the patient, surgery may be indicated especially after the fusion of the apophysis. The excision of the ossicle is the common surgical method, and removal of any prominences on the tibial tuberosity is optional.
